# Peer Review of “Machine Learning–Based Prediction of COVID-19 Mortality With Limited Attributes to Expedite Patient Prognosis and Triage: Retrospective Observational Study”

**DOI:** 10.2196/34082

**Published:** 2021-10-15

**Authors:** Sebastian Daniel Boie

**Affiliations:** 1 Institute of Medical Informatics Charité – Universitätsmedizin Berlin Corporate Member of Freie Universität Berlin, Humboldt-Universität zu Berlin, and Berlin Institute of Health Berlin Germany

**Keywords:** COVID-19, coronavirus, medical informatics, machine learning, artificial intelligence, dimensionality reduction, automation, model development, prediction, hospital, resource management, mortality, prognosis, triage, comorbidities, public data, epidemiology, pre-existing conditions

*This is a peer-review report submitted for the paper “Machine Learning–Based Prediction of COVID-19 Mortality With Limited Attributes to Expedite Patient Prognosis and Triage: Retrospective Observational Study.”*

## Round 1 Review

### General Comments

The paper [1] uses two standard machine learning algorithms to predict mortality of COVID-19 patients, based on a publicly available data set. The data repository contains over 2,600,000 COVID-19–positive samples, of which only a subset of 212 samples were extracted based on the requirement to have full feature availability. A second set of experiments is performed with 5121 samples where symptom information is not required. The performance of the trained logistic regression and random forest algorithms are compared for the data set with 25 features and a reduced data set containing only 7 features. The result is that the reduced feature set leads to higher specificity, sensitivity, accuracy, and area under the curve. An additional result based on the larger data set of 5121 is that age holds a large predictive value.

Many results on mortality prediction of COVID-19 using a range of standard machine learning and advanced deep learning algorithms on larger data samples have entered the literature by now. Since this manuscript uses simple algorithms on a small data set, the strength of this manuscript is neither in the prediction algorithms nor in the relevance of the use case. However, an important line of inquiry is the data quality and the extraction of a small subset of features for good predictive power. To strengthen the results in this area, a more detailed exposition of the feature reduction and comparison with other methods is advisable.

### Specific Comments

#### Major Comments

1. Mutual information as a method for data reduction is not standard to the extent that a single sentence stating that it was used is sufficient. To aid the reader’s understanding and further reproducibility of the article, it should be detailed exactly how this was used. Were the distributions of variables modeled or binned in the mutual information estimate? Were any priors used in the mutual information estimates? The equations, assumptions, and, if used, software packages should be stated in the *Methods* section along with references if the method is not detailed in full in this article.

2. The 7 features that are left after the dimension reduction should be shown and described. Is this a subset of the original feature set or a linear/nonlinear combination of those features?

3. The larger data set of 5121 patients was selected based on the same data completeness requirements apart from symptoms. It is not clear why models for this larger data set were only trained based on the single feature of age. A comparison of the 5121 patients with all features and the 212 with the same features plus symptom data is missing but would give a better estimation of how important symptom data is.

4. Error bars for the relevant test metrics are missing. The sensitivity, specificity, accuracy, and area under the curve are based on 3-fold cross-validation for the models with 25 features and 7 features. Since the effect size is small, error bars should be presented, graphically or numerically, to convince the reader.

5. It would strengthen the results of this paper if the relevance of certain features for the prediction of outcomes would be compared to Estiri et al, who have used a similar methodology on the same use case but on a different data set:

Estiri H, Strasser ZH, Klann JG, et al. Predicting COVID-19 mortality with electronic medical records. *NPJ Digit Med.* 2021;4(15). doi:10.1038/s41746-021-00383-x

6. In the spirit of reproducibility, and since the models are not too complicated, parameters after training should be reported.

#### Minor Comments

7. Since the data repository is continuously updated, the date on which a snapshot was taken should be reported. Ideally, for each experiment, the manuscript should detail exactly which samples were included in training and which were included in testing, because other researchers can directly compare the author’s models and possible alternative models on the same data.

8. It is stated that “Receiver Operator Characteristic curves will be plotted for some classifiers…“ but the plots are missing.

## Round 2 Review

### General Comments

The author has addressed all my previous comments and the manuscript is, from my point of view, sound as far as the application and description of the machine learning methods are concerned.

My only minor comment is that Table 2 should have units (I assume mutual information is measured in bits here).
